# Knowledge about mother–to–child transmission of HIV, its prevention and associated factors among Ethiopian women

**DOI:** 10.7189/jogh.07.020414

**Published:** 2017-12

**Authors:** Tegene Regassa Luba, Zhanchun Feng, Simon Afewerki Gebremedhin, Asfaw N Erena, Abdulsalam MA Nasser, Ghose Bishwajit, Shangfeng Tang

**Affiliations:** 1 School of Medicine and Health Management, Tongji Medical College, Huazhong University of Science and Technology, Wuhan, China; 2 Ministry of Health, Addis Ababa, Ethiopia; 3 School of Public Health, Tongji Medical College, Huazhong University of Science and Technology, Wuhan, China; 4 School of Basic Medical Science, Tongji Medical College, Huazhong University of Science and Technology, Wuhan, China

## Abstract

**Background:**

Poor awareness and knowledge of mother–to–child transmission (MTCT),that accounts for over 90% of new HIV infections among children, might contribute to the HIV epidemics. In Ethiopia, 898 400 children are orphaned due to HIV and AIDS and 200 300 were living with HIV in 2013. The main objective of this study was to examine the knowledge of MTCT of HIV, its prevention (PMTCT) and associated factors among Ethiopian women.

**Methods:**

We conducted a cross–sectional analysis among 16 515 women from the Ethiopian Demographic Health Survey (EDHS) 2011. Chi–square test, univarate and multivariable logistic regression analysis were used to examine the associations of socio–demographic variables with women’s correct knowledge of MTCT and PMTCT, assessed through five specific questions.

**Findings:**

The overall correct knowledge of Ethiopian women about MTCT and PMTCT (correct answers to all the five questions) was very low (34.9%). In the multivariable analysis, residing in urban area (adjusted odds ratio (AOR) = 1.56, 95% CI = 1.35–1.79; *P* < 0.001), having higher education (AOR = 3.25, 95% CI = 2.74–3.86; *P* < 0.001), belonging to higher wealth household (AOR = 1.85, 95% CI = 1.57–2.18; *P* < 0.001), currently in union (AOR = 1.25, 95% CI = 1.12–1.39; *P* < 0.001), occupation (AOR = 1.30, 95% CI = 1.17–1.44; *P* < 0.001) and being exposed to mass media (AOR = 1.55, 95% CI = 1.41–1.70; *P* < 0.001) were strongly associated with women’s correct knowledge of MTCT and PMTCT.

**Conclusion:**

Strategies to improve the knowledge of MTCT and PMTCT in Ethiopia should focus on rural women, emerging regions, the poor, illiterate and unemployed women. Efforts are also needed to involve religious leaders and related organization in the prevention of mother to child transmission of HIV.

Although a remarkable achievement has been made and new HIV infections among children have declined by 50% since 2010 globally [1], HIV remains the major cause of child morbidity and mortality in low resource countries. Among the estimated 36.7 million people living with HIV worldwide in 2015, 1.8 million were children under 15 years of age, and among the estimated 1.1 million people who died of AIDS–related illnesses in the same year, 110 000 were children under 15 years of age [1].

Sub–Saharan Africa is the most affected region, with an estimated 25.6 million people living with HIV in 2015. Among the estimated 2.1 million (150 000 children under 15 years of age) new HIV infections globally in 2015, about 66% occurred in sub–Saharan Africa [2].

Ethiopia is one of the Sub Saharan African countries that are highly affected by HIV and AIDS. An estimated 793 700 people were living with HIV in 2014, with adult prevalence rate of 1.5% [3]. Women account for the larger proportion of people living with HIV and AIDS, with a prevalence rate of 1.9% [4]. In 2013, 78000 women (15–49 ages) were newly infected with HIV [5]. Ethiopia is also among the 22 countries with the highest number of pregnant women living with HIV [6]. In 2013, the number of HIV positive pregnant women was estimated to be 36 467 and about 9493 babies were infected with HIV [7]. According to the 2014 country progress report on HIV response, 200 300 children are living with HIV and there were 898 400 orphans due to HIV and AIDS in Ethiopia [3].

Mother–to–child transmission (MTCT) is the most common route of pediatric HIV infection. It accounts for over 90% of new HIV infections among children [8]. Without intervention, the risk of MTCT ranges from 20% to 45%, but it can be reduced to less than 2%, in non–breastfeeding populations and to 5% or less in breastfeeding populations with effective interventions during the periods of pregnancy, labor, delivery and breastfeeding [9,10]. One of the major problems in preventing mother to child transmission of HIV is the poor awareness and knowledge of the people about MTCT and PMTCT.

While several studies have examined the knowledge on MTCT and PMTCT among pregnant women attending antenatal care [11–16] only one study was conducted among a nationally representative sample of Ethiopian women, to determine whether HIV status and knowledge of mother to child–transmission (MTCT) of HIV were associated with antenatal care (ANC) use [17].

Almost all previous studies investigating MTCT and PMTCT knowledge and associated factors in Ethiopia were done among pregnant women attending antenatal care, and were mainly conducted at health facilities, possibly overestimating the magnitude of the level of knowledge on MTCT and PMTCT of HIV. Thus, in order to carry out a more comprehensive assessment, we examined the knowledge of mother to child transmission of HIV, its prevention and associated factors among a large national sample of Ethiopian women.

## METHODS

### Data source

This study was based on secondary data that were collected for the 2011 Ethiopian Demographic Health Survey (EDHS) which was obtained from USAID–DHS program data sets. EDHS 2011 was conducted by the Ethiopian Central Statistical Agency (ECSA), with a technical assistance from Strategic consulting & communications for a digital world/ICF International and it was part of the worldwide MEASURE DHS project which is funded by the United States Agency for International Development (USAID) [4,18].

Data were collected according to a standard protocol of Demographic and Health Survey (DHS) which had three core survey questionnaires; the Household Questionnaire, the Woman’s Questionnaire and the Man’s Questionnaire. These questionnaires were translated into three major languages of the country—Amharigna, Afan Oromo, and Tigrigna. The questionnaires were pretested in all three languages before the start of field work and respondents were interviewed by the properly trained interviewers. ICF International staff and representatives from other organizations participated in fieldwork monitoring. A quality control team was present in each of 9 regions and 2 city administrations [4].

### Sampling methods

The 2011 EDHS sample was selected using a stratified, two–stage cluster design. In the first stage, the sample included 624 enumeration areas (EAs); 187 in urban areas and 437 in rural areas. In the second stage, a complete listing of households was carried out in each enumeration area. A representative sample of 17 817 households was selected and 17 018 were successfully interviewed, yielding a response rate of 98%. From the sampled households, complete interviews were conducted for 16 515 women aged 15–49 (response rate 95%) and 14 110 men aged 15–59 (response rate 89%), using the relevant questionnaires which were adapted from model survey instruments developed for the MEASURE DHS project.

### Data management

After the relevant variables for the current study were identified, five questions and some key socio–demographic factors were extracted from the large data set. The specific data sets for women were explored and all 16 515 women respondents in the age range selected were included in our analysis.

### Outcome variable

For our analysis, one general question regarding HIV and AIDS (Q1:“Have you ever heard of an illness called HIV and AIDS?”) and 4 other questions (Q2: “the virus that causes AIDS can be transmitted to the baby by breastfeeding?”, Q3: “the virus that causes AIDS can be transmitted to the baby during pregnancy?”, Q.4: “the virus that causes AIDS can be transmitted to the baby during delivery?”) and “Q.5: The Risk of MTCT Can be reduced by taking special drugs (antiretroviral) during pregnancy?” were used to identify the level of respondent’s knowledge on mother to child transmission and its prevention of HIV, with category 1 for ‘yes’ and 0 for ‘no’. All respondents who responded “no” and “don’t know” in the primary data were added in category “0” for the current study, assuming that both were out of awareness. The outcome of interest for this analysis is “women’s correct knowledge of MTCT and PMTCT” and defined as “yes” if the respondent correctly answered all five questions and “no” if the respondent answered any incorrect answer.

### Independent variables

All the relevant socioeconomic factors were considered as independent variables in the analysis of this study and categorized accordingly as follows. Age (15–19, 20–29, 30–39 & 40–49), residence (urban vs rural), marital status (never in union, currently married & formerly married), educational level (no education, primary, secondary & above secondary), Religion (Christians, Muslim, and others), region (agrarian regions, emerging regions and city administrations, wealth index (poorest, poorer, middle, richer and richest), Exposure to mass media (yes, no) and occupation (not working, agricultural and nonagricultural).

Regions were categorized based on the existing structure. Currently, Ethiopia is Administratively structured into nine regional states—Tigray, Affar, Amhara, Oromiya, Somali, Benishangul–Gumuz, Southern Nations Nationalities and Peoples (SNNP), Gambela, and Harari—and two city administrations, Addis Ababa and Dire Dawa administration councils. Hence, four big regions (Oromiya, Amhara, SNNPR and Tigray) were categorized as agrarian regions. Four hard to reach regions, most of them are pastoralist, (Somali, Afar, Benishangul Gumuz and Gambela) were categorized as emerging regions. The capital Addis Ababa, Dire Dawa city and Harari region were categorized in same group. Harari region was categorized with two city administrations based on household’s wealth index and other common similarities as indicated in DHS 2011. The wealth index was categorized based on a standard set of household assets, dwelling characteristics, and ownership of consumer and taken as pre–calculated in the primary data. Watching television, listening radio and reading newspaper at least once a week was considered as being exposed to mass media for the current study.

### Statistical analysis

To identify the background characteristics of respondents in all categories, cross tabulation was done independently. We used chi–square test to check the statistical significance of the associations between the socio–economic variables and a correct knowledge of MTCT and PMTCT. All the variables found to be significant (*P* < 0.001) were entered in to a logistic regression analysis to re–check the associations in univariate, calculate the adjusted odds ratio (AOR, 95% confidence interval. CI) and assess the degree of association between women’s correct knowledge on MTCT & PMTCT and each independent variable in multivariable analysis, adjusting for covariates. Associations were considered significant, if *P*<0.05. All statistical analyses were performed using SPSS software version 22.

## FINDINGS

**Table 1** presents socioeconomic characteristics of the respondents. From the total 16 515 respondents, the majority (67.7%) were from rural area and almost half (48.3%) were from the agrarian regions. Majority of the participants were less than 30 years of age (60.8%), currently married (61.8%) and Christian (61.2%). Half of the participants (50.1%) were illiterate, 48.4% had no formal work and 35% had no access to mass media.

**Table 1:** Distribution of socio–demographic characteristics by knowledge of mother–to–child HIV transmission and prevention of mother–to–child HIV transmission among Ethiopian women

Variables	Number (N = 16515)	Percentage (%)
**Age (years):**		
15–19	3835	23.2
20–29	6207	37.6
30–39	4058	24.6
40–49	2415	14.6
**Residence:**		
Urban	5329	32.3
Rural	11 186	67.7
**Current marital status:**		
Never in union	4413	26.7
Currently married	10 204	61.8
Formerly married	1898	11.5
**Educational level:**		
No education	8278	50.1
Primary	5858	35.5
Secondary	395	8.4
Higher	984	6
**Religion:**		
Christians	10 108	61.2
Muslim	6170	37.4
Others	229	1.4
**Region:**		
Emerging region	4594	27.8
Agrarian region	7984	48.3
City administration and Harari region	3937	23.8
**Wealth index:**		
Poorest	3711	22.5
Poorer	2402	14.5
Middle	2268	13.7
Richer	2505	15.2
Richest	5629	34.1
**Exposure to mass media:**		
No	5766	34.9
Yes	10 736	65
**Occupation:**		
Not working	7992	48.4
Agricultural	3143	19
Non–agricultural	5229	31.7

**Table 2** shows the proportion of respondents who correctly answered to questions identified to evaluate the level of respondent’s knowledge of MTCT and PMTCT. Most of respondents had heard of an illness called HIV and AIDS (96.3%), and 78.1%, 69.3% and 67.1% knew that MTCT could occur through breast feeding, during delivery and during pregnancy respectively. Only 58.9% knew that there are special drugs to avoid HIV transmission to baby. Although a greater proportion of participants answered correctly to individual questions, only 34.9% correctly responded to all five questions.

**Table 2:** Knowledge of respondents on mother–to–child HIV transmission and prevention of mother-to-child HIV transmission among Ethiopian women

Outcome variables	Number of respondents (N = 16515)	Percentage (%)
Ever heard of HIV and AIDS	15 904	96.3
HIV transmitted by breast feeding	12 898	78.1
HIV transmitted during delivery	11 445	69.3
HIV transmitted during pregnancy	11 081	67.1
Drugs to avoid transmission of HIV to baby	9727	58.9
Correct answers to all questions	5648	34.2

The results of χ^2^ test that used to check the statistical significance of the associations between the socio–economic variables and a correct knowledge of MTCT and PMTCT are shown in **Table 3**. All the variables found to be significant (*P* < 0.001) were entered in to a logistic regression analysis to recheck the associations in univariate, calculate the adjusted odds ratio (AOR, 95%CI) and assess the degree of association between women’s correct knowledge on MTCT & PMTCT and each independent variable in multivariate analysis, adjusting for covariates.

**Table 3:** Associations between socio–demographic variables and correct knowledge on mother–to–child HIV transmission and prevention of mother–to–child HIV transmission in chi–square test

Variables	Correct knowledge		Chi–square	*P*
	**N**	**(%)**		
**Total**	**5647**	**34.2%**	54.908	<0.001
**Age (years):**				
15–19 (n = 3835)	1407	36.7		
20–29 (n = 6207)	2236	36.1		
30–39 (n = 4058)	1298	32		
40–49 (n = 2415)	706	29.3		
**Residence:**			18422.225	<0.001
Rural (n = 11 186)	3045	57.2		
Urban (n = 5329)	2602	23.3		
**Current marital status:**			227.661	<0.001
Never in union (n = 4413)	1888	42.8		
Currently married (n = 10204)	3063	30		
Formerly married (n = 1898)	696	36.7		
**Educational level:**			1870.955	<0.001
No education (n = 8278)	1726	20.9		
Primary (n = 5858)	2331	39.8		
Secondary (n = 395)	905	65		
Higher (n = 984)	685	69.7		
**Religion:**			366.629	<0.001
Christians (n = 10 108)	4017	39.8		
Muslim (n = 6170)	1592	25.8		
Other (n = 229)	35	15.3		
**Region:**			805.196	<0.001
Emerging region (n = 4594)	1168	25.5		
Agrarian region (n = 7984)	2410	30.2		
City administration &Harari region (n = 3937)	2069	52.6		
**Wealth index:**			1977.662	<0.001
Poorest (n = 3711)	700	18.9		
Poorer (n = 2402)	513	21.4		
Middle (n = 2268)	516	22.8		
Richer (n = 2505)	733	29.3		
Richest (n = 5629)	3185	56.6		
**Exposure to mass media:**			1013.699	<0.001
No (n = 5766)	1047	18.2		
Yes (n = 10 736)	4598	42.9		
**Occupation:**			332.806	<0.001
Not working (n = 7992)	2427	30.4		
Agricultural (n = 3143)	867	27.6		
Non–agricultural (n = 5229)	2297	44		

Table 4 presents the association between socio–economic variables and correct knowledge of respondents on MTCT and PMTCT in univariate and multivariate analyses. As indicated, the correct knowledge of respondents varied by region and type of residence. Higher proportion of respondents from two city administrations & Harari region (52.6%) and urban dwellers (57.2%) had correct knowledge on MTCT and PMTCT as compared to those from emerging regions (25.5%), agrarian regions (30.2%) and rural area (23.3%).

**Table 4:** Associations between socio–demographic variables and correct knowledge on mother–to–child HIV transmission and prevention of mother–to–child HIV transmission in univariate and multivariate analyses

Variable	Correct knowledge*	Univariate			Multivariate		
	**N (%)**	**OR**	**95% CI**	***P***	**AOR**	**95% CI**	***P***
**Age (Ref = 15–19):**							
15–19 (n = 3835)	1407 (36.7)	Ref	–	–	Ref	–	–
20–29 (n = 6207)	2236 (36.1)	0.97	0.89–1.06	0.511	0.94	0.84–1.05	0.286
30–39 (n = 4058)	1298 (32.0)	0.81	0.74–0.89	<0.001	0.98	0.86–1.12	0.8
40–49 (n = 2415)	706 (29.3)	0.71	0.64–0.80	<0.001	1.02	0.88–1.19	0.766
**Residence (Ref = Rural):**							
Rural (n = 11 186)	2602 (23.3)	Ref	–	–	Ref	–	–
Urban (n = 5329)	3045 (57.2)	4.4	4.11–4.72	<0.001	1.56	1.35–1.79	<0.001
**Marital status (Ref = Never in union):**							
Never in union (n = 4413)	1888 (42.8)	Ref	–	–	Ref	–	–
Currently married (n = 10204)	3063 (30.0)	0.57	0.53–0.62	<0.001	1.25	1.12–1.39	<0.001
Formerly married (n = 1898)	696 (36.7)	0.77	0.69-0.87	<0.001	1.28	1.11-1.48	0.001
**Educational level (Ref = No education):**							
No education (n = 8278)	1726 (20.9)	Ref	-	-	Ref	-	-
Primary (n = 5858)	2331 (39.8)	2.51	2.33-2.70	<0.001	1.77	1.62-1.94	<0.001
Secondary (n = 395)	905 (65.0)	7.03	6.22-7.95	<0.001	2.96	2.56-3.42	<0.001
Higher (n = 984)	685 (69.7)	8.72	7.5310.09	<0.001	3.25	2.74-3.86	<0.001
**Religion (Ref = Other):**							
Christians (n = 10 108)	4017 (39.8)	3.66	2.55–5.26	<0.001	1.85	1.26–2.71	0.002
Muslim (n = 6170)	1592 (25.8)	1.93	1.34–2.78	<0.001	1.34	0.91–1.97	0.134
Other (n = 229)	35(15.3)	Ref	–	–	Ref	–	–
**Region (Ref = emerging region):**							
Emerging region (n = 4594)	1168 (25.5)	Ref	–	–	Ref	–	–
Agrarian region (n = 7984)	2410 (30.2)	1.27	1.17–1.38	<0.001	0.98	0.89–1.08	0.665
City administration &Harari region (n = 3937)	2069 (52.6)	3.25	2.97–3.56	<0.001	1.12	1.00–1.26	0.047
**Wealth index (Ref = Poorest):**							
Poorest (n = 3711)	700 (18.9)	Ref	–	–	Ref	–	–
Poorer (n = 2402)	513 (21.4)	1.17	1.03–1.33	0.017	0.99	0.87–1.13	0.885
Middle (n = 2268)	516 (22.8)	1.27	1.12–1.44	<0.001	1.01	0.88–1.16	0.855
Richer (n = 2505)	733 (29.3)	1.78	1.58–2.0	<0.001	1.27	1.11–1.44	<0.001
Richest (n = 5629)	3185 (56.6)	5.61	5.09–6.19	<0.001	1.85	1.57–2.18	<0.001
**Exposure to mass media (Ref = No):**							
No (n = 5766)	1047 (18.2)	Ref	–	–	Ref	–	–
Yes (n = 10736)	4598 (42.9)	3.38	3.13–3.65	<0.001	1.55	1.47–1.70	<0.001
**Occupation (Ref = Not working):**							
Not working (n = 7992)	2427 (30.4)	Ref	–	–	Ref	–	–
Agricultural (n = 3143)	867 (27.6)	0.87	0.80–0.96	0.003	1.3	1.17–1.44	<0.001
Non–agricultural (n = 5229)	2297 (44.0)	1.79	1.67–1.93	<0.001	1.12	1.03–1.22	0.007

OR – odds ratio, AOR – adjusted odds ratio, CI – confidence interval

*Correct knowledge indicates correct answers to all five questions.

The result of univariate analyses also showed that age, residence, marriage, education, religion, household wealth, exposure to mass media and occupation were significantly associated with women’s correct knowledge of MTCT and PMTCT.

The results of the multivariate analysis that examined the degree of the association between each socioeconomic variable and women’s correct knowledge of MTCT and PMTCT of HIV, adjusting for covariates, are shown in **Table 4**. Results are presented as adjusted odds ratios, 95% confidence intervals and percentages. Overall, place of residence, education level, being in union, religion, exposure to mass media and occupation remained strongly associated with women’s correct knowledge of MTCT and PMTCT of HIV. The association of living in agrarian regions and belonging to Muslim faith with women’s knowledge of MTCT and PMTCT did not reach statistical significance. Logistic regression analysis showed that the higher the education and wealth of respondents, the higher knowledge they have on MTCT and PMTCT of HIV. Women who resided in urban areas, married, with formal work, belonging to Christian faith and those exposed to mass media were more likely to have correct knowledge of MTCT and PMTCT.

## DISCUSSION

This study found out that, though the majority of the participants were aware of MTCT and PMTCT, the level of correct knowledge they had on mother to child transmission of HIV and its prevention was very low (34.9%). This proportion was lower compared to a survey conducted to determine whether HIV status and knowledge of mother to child–transmission (MTCT) of HIV were associated with ANC use in Ethiopia, which indicated an overall prevalence of women’s knowledge on MTCT was 59.9% [17]. It is also lower than the rates observed in facility based studies from Nigeria, Tanzania and Uganda (74.5%, 60%, and 50%, respectively) [19–21].

Residing in urban area, having primary education and above, having higher wealth household, and being exposed to mass media were positively associated with women’s correct knowledge of MTCT and PMTCT. These results support similar findings from Ethiopia, Botswana, Tanzania & Bangladesh [22–26]. The fact that women who resided in urban areas were 1.56 times more likely to have correct knowledge of MTCT and PMTCT than rural women could be due to better access of urban residents to health information and education through electronic and social medias. Although we didn’t analyze the distance between residence and health facilities, the accessibility of health facilities in urban area might be another reason as it is the place where health educations are given to women during ANC and related services. Further study is needed to examine the impact of distance between residence and health facilities, and the role of social medias in raising the awareness and knowledge of women on MTCT and PMTCT.

Having primary education and above, having higher level of wealth quintile and being exposed to mass media were strongly associated with women’s correct knowledge of MTCT and PMTCT. For example women who had higher education level and women from the richest household were 3.25 and 1.85 times more likely to have correct knowledge of MTCT and PMTCT compared to the illiterate and poorest women, respectively. The potential explanation might be that educated women have more access to different health information and can capture the content easily. The low level of knowledge on MTCT and PMTCT among the poor women might be due to less access to health services and health information related to MTCT and PMTCT.

Women with access to mass media (watching television, listening radio and reading newspaper at least once a week) were 1.55 times more likely to have correct knowledge of MTCT and PMTCT than those with no access. This finding is consistent with the studies from Kenya and Tanzania [25,27]. Addressing the illiterate and poor women in the PMTCT services and reaching them with target oriented MTCT and PMTCT messages through different mass media is needed to achieve elimination of MTCT.

Women’s correct knowledge of MTCT and PMTCT was positively associated with occupation. This finding is in agreement with previous studies from Ethiopia and Kenya [16,28]. A possible explanation is that women who have formal work have better access to health information and education at their work places and through different media compared to unemployed women. Marriage was also positively associated with women’s correct knowledge of MTCT and PMTCT. The fact that the women currently in union were 1.25 times more likely to have correct knowledge of MTCT and PMTCT compared to the women never in union could indicate that married woman obtain health information at health facilities during their visit for ANC and related services. Moreover, married women may share information about MTCT and PMTCT with their male partners. Conducting further studies on male’s knowledge about MTCT and PMTCT and its impact on male involvement in PMTCT programs is valuable in these regards.

The results also show that Christian women had higher levels of knowledge on MTCT and PMTCT. Women who belonged to Christian faith were 1.85 times more likely (*P* = 0.002) to have correct knowledge of MTCT and PMTCT. This fact may indicate the active engagement of churches, religious leaders and related organizations in combating HIV/AIDS in general and in promoting PMTCT programs in particular. Previous studies from Nigeria and Uganda also indicated that religious leaders and organizations can play an important role in raising the awareness of the people to combat HIV and AIDS epidemic [29,30].

The finding of current study regarding women from emerging regions being less likely to have correct knowledge of MTCT and PMTCT compared to those reside in city administration and Harari region is consistent with similar studies conducted in city administrations and emerging region of Ethiopia. For instance, the study conducted in Addis Ababa (city administration) shows 89.8% and 76.8% of respondents having knowledge of MTCT and PMTCT respectively [31]. However, the result of a study conducted in Assosa town (emerging region) found out only 57.5% and 17.4% of respondents had knowledge about MTCT and PMTCT of HIV respectively [32]. Poor access and utilization of PMTCT due to limited access of infrastructures like health facilities, schools, roads, distance from the central government and limited access of media coverage in emerging regions could be possible reasons. The findings of this study have implications for policy makers and all concerned bodies enrolling women who live in emerging regions in PMTCT programs. Hence, more efforts and holistic approaches are needed to intensify health education and all PMTCT related services particularly in these regions.

This study has a number of limitations. First, the primary data were collected for 2011 EDHS and therefore it may not reflect adequately the current situation. Second, as this is a cross–sectional study, it is not possible to make causal inferences and determine the temporal nature of the associations. Third, some variables which may have effect on women’s knowledge of MTCT and PMTCT; like ANC services, distance to reach health facilities, HIV counseling and testing, misconceptions of HIV and AIDS were not analyzed in the current study.

However, as it was conducted on national sample of Ethiopian Women, the findings would contribute a lot to interventions aimed at increasing women’s awareness and knowledge of mother to child transmission of HIV, its prevention and associated factors. The findings of this study have important implications for policy makers and other concerned bodies. It can also serve as a benchmark for those who want to make a further study to identify the knowledge of general population about MTCT and PMTCT in Ethiopia.

## CONCLUSION

The overall level of knowledge of Ethiopian women about MTCT and PMTCT was very low. Our study found out that educated women, women who resided in urban area, women of rich household, women who belonged to Christian faith and those who were exposed to mass media were relatively at a better position to have correct knowledge of MTCT and PMTCT. Women who got married or being in union and had formal work were also more likely to have better knowledge of MTCT and PMTCT. Strategies to improve the knowledge of MTCT and PMTCT in Ethiopia should focus on women who live in rural area, emerging regions, the poor, illiterate and unemployed women. Lastly, efforts are needed to involve religious leaders and related organizations in the prevention of mother to child transmission of HIV across the country.

## References

[1] UNAIDS. HIV and AIDS global statistics. 2016. Available: http://www.unaids.org/en/resources/fact–sheet. Accessed: 30 October 2016.

[2] US Department of Health & Human Services. HIV and AIDS global statistics. 2016.Available: https://www.aids.gov/hiv-aids-basics/hiv-aids-101/global-statistics/. Accessed: 29 December 2016.

[3] FHAPCO. Country progress reports on the HIV response. 2014. Available: http://www.unaids.org/en/regionscountries/countries/ethiopia. Accessed: 29 December 2016.

[4] ECSA. Ethiopian demographic and health survey. 2011. Available: http://dhsprogram.com/publications/publication-fr255-dhs-final-reports.cfm. Accessed: 31December 2016.

[5] UNAIDS. Gaps report on the Progress toward achieving the goals of the Global Plan to wards the elimination of new HIV infections among children by 2015 and keeping their mothers alive. 2014. Available: http://www.unaids.org/en/resources/documents/2014/JC2681_2014-Global-Plan-progress.Accessed: 2 January2017.

[6] WHO. Global update on HIV treatment. 2013. Available: http://www.unaids.org/en/resources/documents/2013/20130630_treatment_report. Accessed: 2 January 2017.

[7] Ministry of Health. Health and Health related indicators. 2013. Available: https://www.dktethiopia.org/sites/default/files/PublicationFiles/Health%20and%20Health%20Related%20Indicators%202005%20E.C.pdf. Accessed: 3January 2017.

[8] WHO. Guidance on global scale-up of the prevention of mother to child transmission of HIV. 2007, Available: http://www.who.int/hiv/pub/toolkits/PMTCT9789241596015_eng.pdf. Accessed: 15 January 2017.

[9] WHO. PMTCT strategic vision 2010–2015: preventing mother-to-child transmission of HIV to reach the UNGASS and Millennium Development Goals. 2010. Available: http://www.who.int/hiv/pub/mtct/strategic_vision/en/. Accessed: 11January 2017.

[10] WHO. Mother-to-child transmission of HIV. 2016. Available: http://www.who.int/hiv/topics/mtct/en /. Accessed: 5January 2017.

[11] Deressa W, Seme A, Asefa A, Teshome G, Enqusellassie F (2014). Utilization of PMTCT services and associated factors among pregnant women attending antenatal clinics in Addis Ababa, Ethiopia.. BMC Pregnancy Childbirth.

[12] Abajobir AA, Zeleke AB (2013). Knowledge, attitude, practice and factors associated with prevention of mother-to-child transmission of hiv/aids among pregnant mothers attending antenatal clinic in Hawassa Referral Hospital, South Ethiopia.. J AIDS Clin Res.

[13] Tesfaye G, Tufa B, Likisa J, Alebachew M, Temesgen G, Dinsa H (2014). Knowledge, attitude and practice towards PMTCT of HIV among women attending Ambo Hospital ANC Clinic, West Ethiopia.. J AIDS Clin Res.

[14] Hamdela B, Zekiewos F, Workneh T (2014). Knowledge on mother to child transmission and utilization of services designed to prevent mother to child transmission of HIV/AIDS among pregnant women in Hossana Town, Southern Ethiopia.. J AIDS Clin Res.

[15] Gebre Y, Astede F (2012). Assessment of knowledge, attitude and practice of HIV prevention of mother to child among pregnant women attending Ayder referral hospital, Mekelle, Ethiopia.. IJMBS.

[16] Asefa A, Beyene H (2013). Awareness and knowledge on timing of mother-to-child transmission of HIV among antenatal care attending women in Southern Ethiopia: a cross sectional study. 2013.. Reprod Health.

[17] Sahlu I, Howe CJ, Clark MA, Marshall BDL (2014). HIV status, knowledge of mother-to child transmission of HIV and antenatal care use among Ethiopian women.. J Epidemiol Glob Health.

[18] International ICF. Ethiopian Demographic health survey (EDHS). 2012. Available: http://www.measuredhs.com. Accessed: 20 September 2016.

[19] Adelaja-Lamina A (2012). A survey of awareness and knowledge of mother-to-child transmission of HIV in pregnant women attending Olabisi Onabanjo University Teaching Hospital, Sagamu, Nigeria.. Open J Obstet Gynecol.

[20] Wangwe PJ, Nyasinde M, Charles DS (2013). Counselling at primary health facilities and level of knowledge of antenatal attendees and their attitude on prevention of mother to child transmission of HIV in Dar-es Salaam, Tanzania.. Afr Health Sci.

[21] Byamugisha R, Tumwine JK, Ndeezi G, Karamagi CAS, Tylleskär T (2010). Attitudes to routine HIV counseling and testing, and knowledge about prevention of mother to child transmission of HIV in eastern Uganda: a cross-sectional survey among antenatal attendees.. J Int AIDS Soc.

[22] Malaju MT, Getu DA (2012). Determinant factors of pregnant mothers’ knowledge on mother to child transmission of HIV and its prevention in Gondar tow, North West Ethiopia.. BMC Pregnancy and Childbirth.

[23] Birhane T, Gizachew AT, Kefyalew AA, Abel FD (2015). Knowledge of pregnant women on mother-to-child transmission of HIV in Meket District, Northeast Ethiopia.. Journal of Pregnancy.

[24] Majelantle RG, Keetile M, Bainame K, Nkawana P (2014). Knowledge, opinions and attitudes towards HIV and AIDS among youth in Botswana.. J Glob Econ.

[25] Haile ZT (2016). Teweldeberhan Ak, Chertok IRA. Correlates of women’s knowledge of mother-to-child transmission of HIV and its prevention in Tanzania: a population-based study. AIDS Care.

[26] Mondal MN, Hoque N, Chowdhury RK, Hossain MS (2015). Factors associated with misconceptions about HIV transmission among ever-married women in Bangladesh.. Jpn J Infect Dis.

[27] Granberg C. Socioeconomic and sociodemographic differentials in pregnant women’s knowledge on prevention of mother-to-child HIV transmission in Kenya: A cross-sectional study from 2008-09 KDHS. 2015. Available: http://urn.kb.se/resolve?urn=urn:nbn:se:uu:diva-253890. Accessed: 1 October 2016.

[28] Kei RM, Ndwiga T, Okong’o SO, Mburu SN (2015). Knowledge and attitude on prevention of mother to child transmission of HIV among pregnant women attending antenatal clinic at Kisii Level Five Hospital in Kisii County, Kenya.. Int J Trop Dis Health.

[29] Olugbenga-Bello A, Adebimpe WO, Osundina FF, Abdulsalam ST (2013). Perception on prevention of mother-to-child-transmission (PMTCT) of HIV among women of reproductive age group in Osogbo, Southwestern Nigeria. Int J Womens Health.

[30] Marshall K, Keough L. Mind, heart and soul in the fight against poverty. 2004. Washington, DC: World Bank. Available. http://documents.worldbank.org/curated/en/220251468762875492/Mind-heart-and-soul-in-the-fight-against-poverty. Accessed: 1 October 2016.

[31] Solomon J, Tilahun T (2005). Knowledge and attitude towards mother to child transmission of HIV and its prevention among postnatal mothers in Tikur Anbessa and Zewditu Memorial Hospitals Addis Ababa.. Ethiop J Health Dev.

[32] Abtew S, Awoke W, Asrat A (2016). Knowledge of pregnant women on mother-to-child transmission of HIV, its prevention, and associated factors in Assosa town, Northwest Ethiopia.. HIV AIDS (Auckl).

